# Evaluating the diagnostic accuracy of TpN17 and TmpA recombinant proteins in syphilis detection: a phase II study

**DOI:** 10.3389/fmicb.2024.1348437

**Published:** 2024-02-27

**Authors:** Ângelo Antônio Oliveira Silva, Ayla Araújo Lima, Larissa Carvalho Medrado Vasconcelos, Rosângela Andrade Almeida, Natália Erdens Maron Freitas, Talita Andrade Oliva, Miralba Freire de Carvalho Ribeiro da Silva, Fabricio Klerynton Marchini, Nilson Ivo Tonin Zanchin, Isadora Cristina de Siqueira, Fred Luciano Neves Santos

**Affiliations:** ^1^Advanced Public Health Laboratory, Gonçalo Moniz Institute (IGM) Foundation Oswaldo Cruz (Fiocruz-BA), Salvador, Brazil; ^2^Salvador University (UNIFACS), Salvador, Brazil; ^3^State Center for Diagnosis, Assistance, and Research (CEDAP), Salvador, Brazil; ^4^Molecular Biology Institute of Paraná, Curitiba, Brazil; ^5^Laboratory for Applied Science and Technology in Health, Carlos Chagas Institute (ICC) Oswaldo Cruz Foundation (Fiocruz-PR), Curitiba, Brazil; ^6^Structural Biology and Protein Engineering Laboratory, Carlos Chagas Institute (ICC) Oswaldo Cruz Foundation (Fiocruz-PR), Curitiba, Brazil; ^7^Integrated Translational Program in Chagas Disease from Fiocruz (Fio-Chagas), Oswaldo Cruz Foundation (Fiocruz-RJ), Rio de Janeiro, Brazil; ^8^Laboratory of Investigation in Global Health and Neglected Diseases, Gonçalo Moniz Institute (IGM) Oswaldo Cruz Foundation (FIOCRUZ-BA), Salvador, Brazil

**Keywords:** syphilis, recombinant proteins, immunodiagnosis, TpN17 protein, TmpA protein, ELISA, treponemal test

## Abstract

Syphilis is a sexually transmitted infection (STI) caused by the spiral bacterium *Treponema pallidum*. Diagnosis is based on epidemiology, clinical and serology, but serodiagnosis is challenging because distinct clinical forms of the infection may influence serological performance. Several recombinant *Treponema pallidum*-proteins have already been tested for syphilis diagnosis and they are critical to achieve high accuracy in serological testing. A total of 647 samples were included in the study: 180 *T. pallidum*-positive samples, 191 *T. pallidum*-negative samples and 276 sera from individuals infected with unrelated diseases. The diagnostic potential was validated by analysis of ROC curves. For the indirect ELISA, TpN17 (100%) and TmpA (99%) showed excellent AUC values. Sensitivity values were 97.2% for TpN17 and 90.6% for TmpA, while specificity was 100% for both molecules. According to the clinical phase, TmpA ranged from 84% to 97%, with the highest value for secondary syphilis. TpN17 was 100% sensitive for the primary and secondary stages and 93.2% for recent latent syphilis. All clinical phases achieved 100% specificity. Accuracy values showed that TmpA (> 95%) and TpN17 (> 98%) presented high diagnostic accuracy for all clinical stages of syphilis. Cross-reactivity was only observed in one sample positive for Chagas disease (1.5%), when TpN17 was evaluated. On the other hand, TmpA showed reactivity for two samples positive for Chagas disease (3.1%), one sample positive for HBV (1.25%), two samples positive for HIV (9.5%) and one sample positive for HTLV (1.6%). The TmpA antigen’s performance was evaluated in multiple studies for syphilis diagnosis, corroborating our findings. However, TpN17 sensitivity values have ranged in other studies. According to clinical stages of the infection, our findings obtained close performance values.

## 1 Introduction

Syphilis, a sexually transmitted infection (STI), is caused by the spirochete bacterium *Treponema pallidum*, subspecies pallidum (order Spirochaetales). It manifests in multiple clinical stages with distinct characteristics (Larsen et al., 1995). Primary syphilis presents as a painless, typically solitary, clean-based, indurated ulcer (chancre) at the site of bacterial replication (LaFond and Lukehart, 2006; Hook, 2017; Peeling et al., 2017; O’Byrne and Macpherson, 2019). About two to 12 weeks after sexual contact, it progresses to secondary syphilis, characterized by diffuse skin rashes and mucosal lesions (Hook, 2017; Peeling et al., 2017; O’Byrne and Macpherson, 2019). If left untreated, syphilis may advance through asymptomatic stages (recent and late latent) and lead to symptomatic forms such as tertiary syphilis, which is characterized by slowly progressive systemic symptoms, including necrotic granulomatous lesions, neurosyphilis, and cardiovascular syphilis (Hook, 2017; Arando Lasagabaster and Otero Guerra, 2019). Congenital syphilis occurs when infected pregnant women transmit the infection during childbirth or breastfeeding (Singh and Romanowski, 1999; Giacani and Lukehart, 2014).

In recent decades, there has been an alarming increase in reported syphilis cases. Approximately 6.3 million cases are reported annually among individuals aged 15 and 49 years worldwide. The global incidence rate was 1.7 cases per 1,000 women and 1.6 per 1,000 men in 2016 and prevalence ranges from 0.1 to 1.6% across different regions from 2012 to 2016 (Rowley et al., 2019). Only Brazil, there were 167,523 cases of acquired syphilis and 74,095 cases of syphilis in pregnant women reported in 2021. Additionally, 27.1 cases per 1,000 live births were detected, along with 27,019 cases of congenital syphilis, yielding an incidence rate of 9.9 cases per 1,000 live births (Ministério da Saúde, 2022).

Laboratory diagnosis of syphilis involves serological tests, including treponemal (TTs) and non-treponemal (NTTs). These tests are employed in either a traditional approach (NTTs for screening and TTs for confirmation) or a reverse algorithm (TTs for screening, NTTs for confirmation of positive samples, and secondary TT testing in case of discordant results) (Clyne and Jerrard, 2000). NTTs such as VDRL (venereal disease research laboratory) and RPR (rapid plasma reagin) are used to qualitatively and quantitatively detect anticardiolipin antibodies produced between the 1st and 2nd weeks after the appearance of the indurated ulcer (chancre), for both screening and monitoring therapeutic progress (Larsen et al., 1995; LaFond and Lukehart, 2006). TTs are necessary for the qualitative detection of IgG or IgM anti-*T. pallidum* antibodies, offering automated methodologies, rapid screening, and high-precision quantification for multiple samples with high precision (Clyne and Jerrard, 2000; Forrestel et al., 2020), with superior diagnostic performance compared to NTTs (Park et al., 2019).

The performance of ELISA in syphilis diagnosis varies depending on the antigens used for detecting anti-*T. pallidum* antibodies (Liu et al., 2019). Antigenic matrices can incorporate lysates of *T. pallidum* or recombinant antigens, either isolated or in combination (Larsen et al., 1995). In a phase I study, TpN15, TpN17, TpN47, and TmpA recombinant proteins exhibited superior diagnostic performance (Silva et al., 2020). TpN17 and TmpA showed the most promising results and advanced to a phase II study. Several other studies have explored these proteins in the development of serological tests, seeking precision, rapidity, and cost-effectiveness (Young et al., 1998; Sato et al., 1999; Xu et al., 2016). However, limited research involving these proteins has been conducted in Brazil, despite the high incidence of syphilis. Therefore, our study aims to optimize indirect ELISA for assessing the diagnostic potential of TpN17 and TmpA proteins using samples from serologically positive individuals in Brazil.

## 2 Materials and methods

### 2.1 Recombinant proteins synthesis

We followed the method previously described (Silva et al., 2020) for recombinant protein synthesis. In brief, we acquired synthetic genes of the bacterium *T. pallidum* subspecies pallidum Nichols strain from a commercial supplier (GenScript, Piscataway-NJ, United States). These synthetic genes, purchased in pUC57, were subcloned in-house into the pET28a expression vector, and expression was carried out in *Escherichia coli* strain BL21-Star (DE3). Bacterial cells were initially incubated for 16 h at 37°C in Luria-Bertani broth containing kanamycin (50 μg/mL). The culture was then diluted at 1:20 in fresh medium and incubated at 37°C until an optical density ranging from 0.6 to 0.8 was reached, measured at 600 nm (OD600). Expression was induced by adding IPTG (isopropyl β-D-1-thiogalactopyranoside) to a final concentration of 500 μM and incubating for 4 h at 37°C. Bacterial cell disruption was achieved using either a microfluidizer processor (Microfluidics Model M-110 L, Hyland Scientific, Stanwood-WA, United States) or chemical methods. The resulting recombinant proteins were purified through affinity and ion exchange chromatography. Proteins were quantified using a fluorometric assay (Qubit12.0, Invitrogen Technologies, Carlsbad-CA, United States), and purity was confirmed through SDS-PAGE stained with CBB-G250 (Laemmli, 1970).

### 2.2 Sampling

The sample size calculation, conducted with a 95% confidence interval, expected sensitivity and specificity of 99%, and an absolute error of 1.5%, using the OpenEpi program^1^ (Dean et al., 2013), determined that we needed 169 *T. pallidum*-positive samples (TpP) and 169 *T. pallidum*-negative samples (TpN) to conduct this study. Seven hundred two samples were deemed eligible. Following sera characterization, we excluded 65 samples, leaving 647 samples for analysis.

Panel 1: consisted of 180 *T. pallidum*-positive samples obtained from CEDAP, Bahia State Health Department, between April 26, 2021, and November 12, 2021. These samples were obtained through serological tests (rapid immunochromatographic test and VDRL) and clinical examinations for syphilis conducted by healthcare professionals at the reference center. Of these, 30 were from primary syphilis, 77 from secondary syphilis, and 73 from early latent syphilis. We obtained 191 *T. pallidum*-negative samples from the Foundation of Hematology and Hemotherapy of the State of Bahia (HEMOBA) from December 2017 to April 2019, stored in our biorepository.

Panel 2: contained 276 samples from individuals with unrelated diseases, previously diagnosed using parasitological or serological methods. These samples were obtained from HEMOBA and the Foundation of Hematology and Hemotherapy of the State of Pernambuco (HEMOPE) and included chronic Chagas disease (*n* = 65), hepatitis B virus (*n* = 80), hepatitis C virus (*n* = 49), HIV-1/2 (*n* = 21), and HTLV-1/2 (*n* = 61). Sample selection for cross-reactivity assessment was based on confirmed disease positivity and concurrent negativity for syphilis in the chemiluminescence assay conducted at the blood bank.

All serum samples from panel 1 and 2 underwent serological retesting for *T. pallidum* antibodies using one non-treponemal (NTT): VDRL test kit (Wiener Lab, Rosario, Argentina), and two treponemal tests (TTs): anti-*Treponema pallidum* IIFT (IgG) test (Euroimmun Medizinische Labordiagnostika AG, Lübeck, Germany), and recombinant ELISA v.4.0 test (IgG) (Wiener lab., Rosario, Argentina). All testing was performed in accordance with the manufacturer’s instructions.

A sample was considered positive when it tested positively in two treponemal tests (ELISA and FTA-ABS) and one non-treponemal test (VDRL). Conversely, it was deemed negative if it did not show positivity in two treponemal tests (ELISA and FTA-ABS). In cases of equivocal or positive results, samples were subjected to a non-treponemal test (VDRL). Samples with volume less than 50 μL and those with uncertain or undefined serological evaluation for syphilis were not included in this study. Each sample was assigned a unique identifier code to ensure a blinded analysis.

### 2.3 Indirect ELISA

Standardization followed the checkerboard titration as previously described (Silva et al., 2020) and was optimized for this study. In summary, 100 ng/well of TpN17 and 200 ng/well of TmpA, in coating buffer (0.05 M carbonate–bicarbonate, pH 9.6), were added to transparent flat-bottom polystyrene microplates (Microtest Plate 96 Well, F—Sarstedt, Germany). These were incubated at room temperature (RT) for 15 min. Subsequently, the microplates were blocked and stabilized with 100 μL/well of WellChampion^™^ (Ken-En-Tec Diagnostics A/S, Taastrup, Denmark) synthetic blocking buffer for 15 min, following the manufacturer’s instructions. Afterward, the microplates were emptied, inverted, and placed in an oven at 37°C for 90 min for drying (MARCONI^®^ MA032, São Paulo-SP, Brazil). They were then washed once in 0.75% Tween-20 plus PBS buffer (PBS-T). Serum samples were loaded at 1:25 in PBS-T, adding 100 μL/well. After incubation for 30 min at 37°C, the microplates were washed in PBS-T to remove any unbound antibodies. HRP-conjugated secondary antibody (SIGMA SAB3701283; Sigma-Aldrich, San Luis, Missouri, EUA; lot RI39338), diluted at 1:10.000 (TmpA and TpN17) in PBS-T, was added to each well, followed by incubation for 30 min at 37°C and subsequent washing with PBS-T. For the detection of the immunocomplexes, 100 μL of TMB Plus solution (tetramethyl-benzidine; Ken-En-Tec Diagnostics A/S, Taastrup, Denmark) was added to each well. After 15 min of incubation at RT in the dark, the reaction was halted with 50 μL of 0.3 M H2SO4, and absorbance was measured at 450 nm in a microplate spectrophotometer (SPECTRAmax 340PC1, California, United States).

### 2.4 Statistical analysis

Statistical analysis was conducted using scatter computer graphic software (GraphPad Prism version 9, San Diego-CA, United States). The variables were analyzed using descriptive measures, including arithmetic and geometric means and standard deviation (SD). The geometric means were calculated with a 95% confidence interval (CI). Data normality was assessed using the Shapiro–Wilk test. The Wilcoxon–Mann–Whitney or Kruskal–Wallis test was employed when the null hypothesis was rejected. When the normality was confirmed, the Student’s *t*-test was used. All conclusions were drawn at a significance level of *p* < 0.05. The cut-off (CO) values for TpN17 were determined based on the area under the ROC curve (AUC) (TpN17), while for TmpA, they were established using the mean of the negative values plus 2 standard deviations (X̄ NEG + 2SD). Results were normalized by calculating the reactivity index (RI), denoting the ratio between the OD of the samples and the CO. Samples with RI ≥ 1.0 were considered positive, RI < 1.0 negative, and RI = ±10% of 1.0 classified as inconclusive (gray zone). To assess global accuracy for each antigen, we calculated areas under the ROC curve (AUC), interpreted as outstanding (1.0), elevated (0.82–0.99), moderate (0.62–0.81), or low (0.51–0.61) (Swets, 1988). We determined and compared ELISA performance parameters regarding sensitivity (Sen), specificity (Spe), accuracy (Acc), positive (LR+) and negative (LR-) likelihood ratio, and diagnostic odds ratio (DOR). We established the agreement strength between the standard tests and ELISA through Cohen’s Kappa (*κ*), interpreted as follows: 1.0 ≤ *κ* ≥ 0.81 (almost perfect agreement), 0.80 ≤ *κ* ≥ 0.61 (substantial agreement), 0.60 ≤ *κ* ≥ 0.41 (moderate agreement), 0.40 ≤ *κ* ≥ 0.21 (fair agreement), 0.20 ≤ *κ* ≥ 0 (slight agreement), or *k* = 0 (poor agreement) (Landis and Koch, 1977). A checklist is provided according to the Standards for the Reporting of Diagnostic accuracy studies (STARD) guidelines.

## 3 Results

### 3.1 Sera characterization

**Figure 1:**
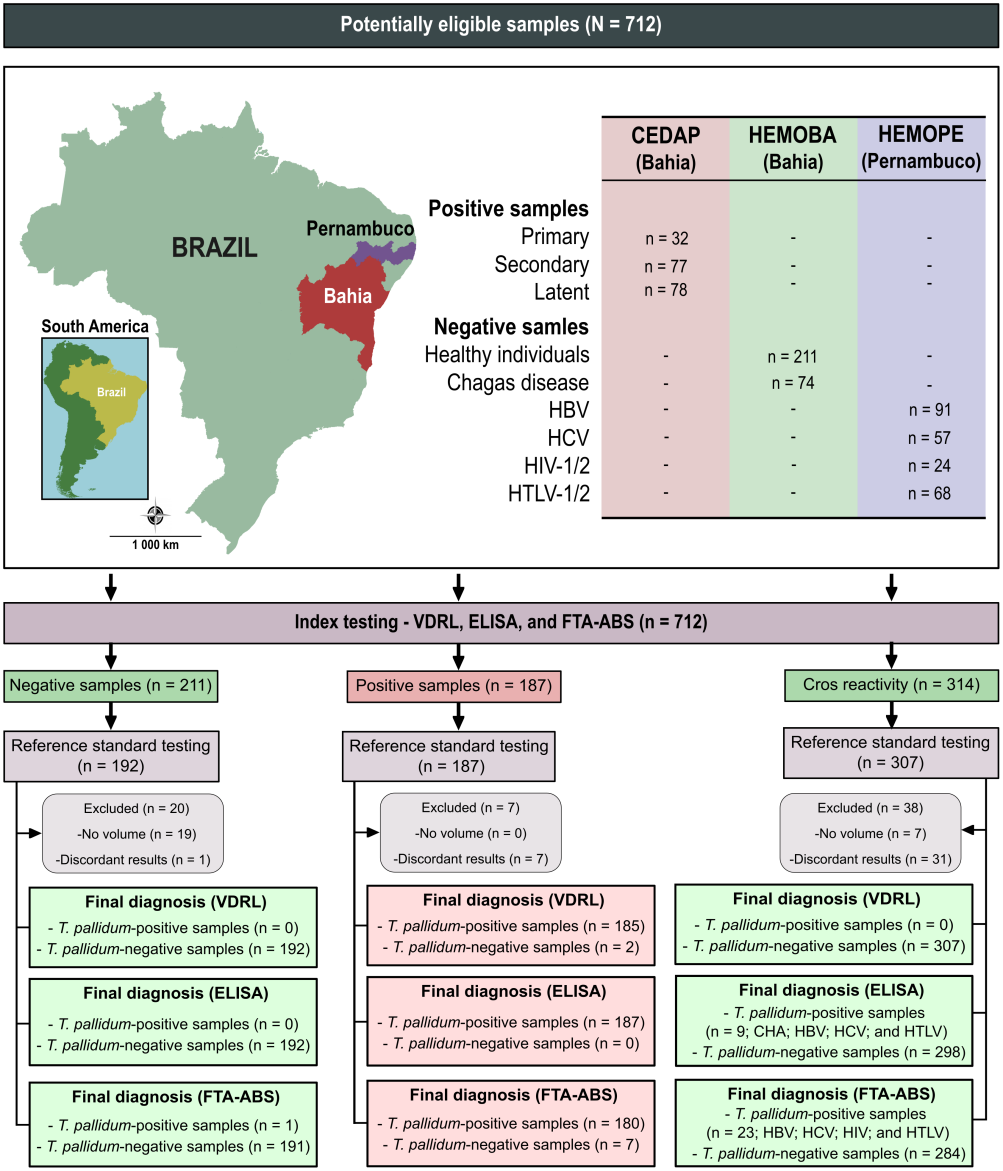
Sera characterization from *Treponema pallidum*-positive and negative samples tested with VDRL, ELISA, and IIFT. NR, non-reagent; R, reagent; VDRL, Venereal Disease Research Laboratory; ELISA, enzyme-linked immunosorbent assay; IIFT, indirect immunofluorescence—IgG.

In this study, we employed a total of 712 serum samples (Figure 1). Initially, all samples underwent re-assessment using non-treponemal (VDRL) or treponemal (ELISA and FTA-ABS) tests. Samples returning discordant results or having insufficient volume were excluded (*n* = 65; 9.1%). Following characterization, 180 positive samples were included, and seven were excluded due to discordant results (seven reagent samples for the ELISA, two non-reagent samples for the VDRL and seven reagent samples for the IIFT). Among negative samples, 191 were confirmed as negatives, with only one sample testing positive for IIFT. Regarding positive samples for unrelated diseases, 276 yielded negative result, while 31 showed discordant results (ELISA: eight reagent samples but non-reactive in IIFT; IIFT: 22 reagent samples but non-reactive in ELISA; VDR: one non-reactive sample but reagent in ELISA and IIFT).

### 3.2 ELISA performance

**Figure 2:**
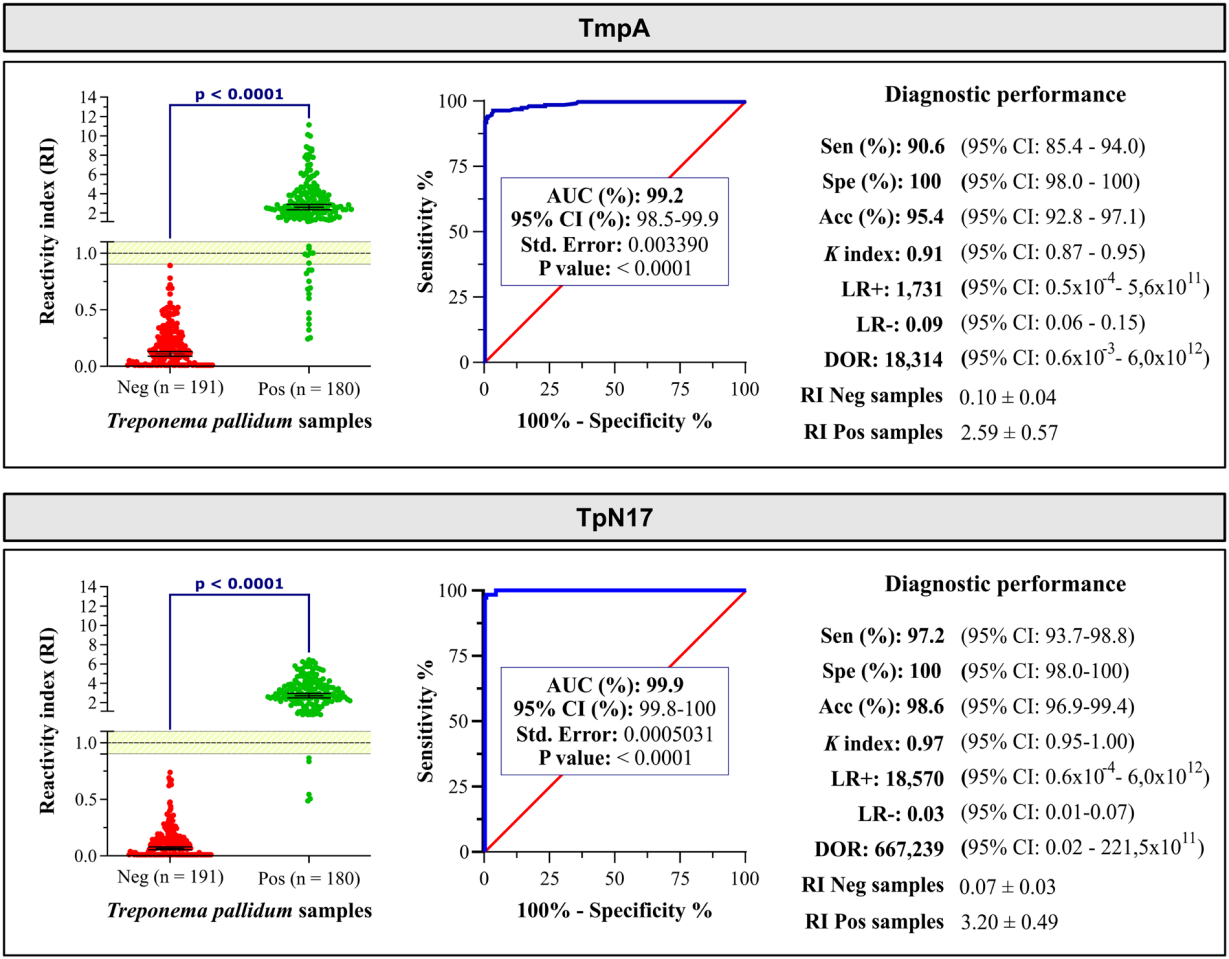
The reactivity index and diagnostic performance parameters of the TmpA and TpN17 for *Treponema pallidum*-positive and negative serum samples. The cut-off is set at the reactivity index value = 1.0 and the shaded area represents the gray zone (RI = 1.0 ± 0.10). Horizontal lines and numbers represent the geometric means (±95% CI) for each result group. AUC, area under curve; Sen, sensitivity; Spe, specificity; Acc, accuracy; LR+, positive likelihood ratio; LR−, negative likelihood ratio; DOR, diagnostic odds ratio; Neg, negative; Pos, positive; ĸ index, Cohen’s Kappa index.

Both TmpA and TpN17 exhibited exceptional discrimination between positive and negative samples for syphilis, with AUC values of 99.2% for TmpA and 99.9% for TpN17. The TmpA and TpN17 *T. pallidum*-recombinant proteins achieved sensitivities of 90.6 and 97.2%, respectively. TpN17 identified five (2.8%) false-negative samples, while TmpA identified 17 (9.4%) false negatives, affecting the diagnostic sensitivity. The maximum specificity value was obtained for both proteins, with 100% for TmpA and TpN1. Accuracy values were 95.4% for TmpA and 98.7% for TpN17 (Figure 2 and Supplementary Table S1).

According to the positive likelihood ratio (LR+), both TpN17 and TmpA exhibited a significant increase (LR > 10) in the probability of a positive result for a person with syphilis compared to a negative person, with values of 1,857.92 and 1,730.52, respectively. The negative likelihood ratio (LR-) showed a significant decrease (LR < 0.1), with values of 0.03 (TpN17) and 0.09 (TmpA), indicating low probability of a negative result for a person with syphilis compared to a negative person. DOR values demonstrated the high diagnostic performance of botht TpN17 and TmpA, with values of 66,850 and 18,313.53 times the chance the test being positive for a person with syphilis compared to a negative person. Kappa values indicated almost perfect agreement (TmpA and TpN17) with the reference tests (Table 1).

**Table 1:** Kappa values for TpN17 and TmpA compared to the gold standard.

Result	Reverse algorithm		*κ* (95% CI)
	Positive	Negative	
TpN17			0.97 (0.97–1.00)
Positive	177	0	
Negative	3	191	
Total	180	191	
TmpA			0.91 (0.88–0.96)
Positive	165	0	
Negative	15	191	
Total	180	191	

*κ*, Cohen’s kappa; CI, confidence interval.

**Figure 3:**
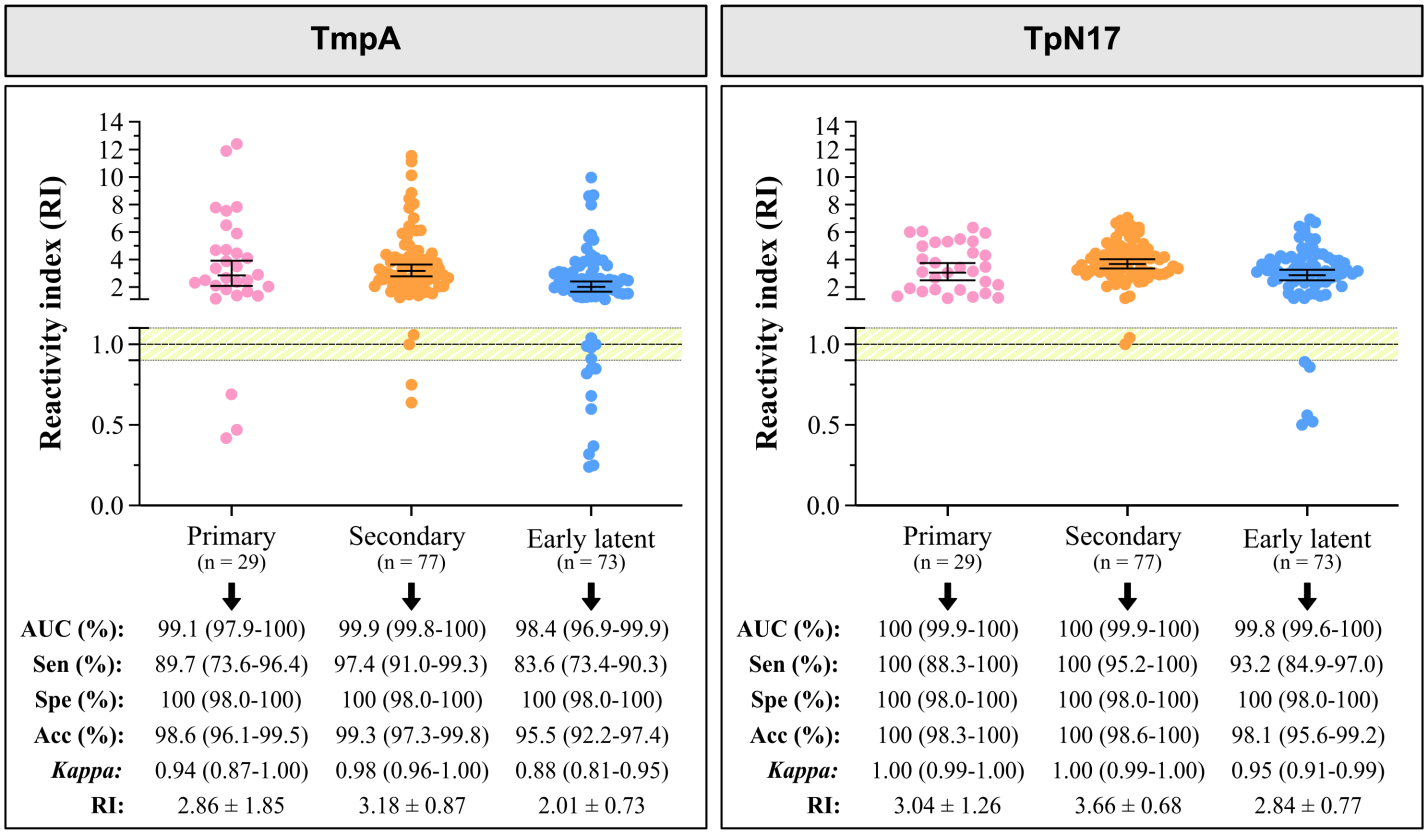
The reactivity index and diagnostic performance parameters of the TmpA and TpN17, stratified by clinical stage, for *Treponema pallidum*-positive and negative serum samples. The cut-off is set at the reactivity index value = 1.0 and the shaded area represents the gray zone (RI = 1.0 ± 0.10). Horizontal lines and numbers represent the geometric means (±95% CI) for each result group. AUC, area under curve; Sen, sensitivity; Spe, specificity; Acc, accuracy; RI, reactivity index.

Regarding the inconclusive zone (RI = 1.0 ± 10%), few positive samples yielded inconclusive results: three (1.7%) inconclusive results for TpN17 testing and seven (3.9%) for TmpA (Figure 3). No negative sample fell within the inconclusive space when tested using TmpA. However, one negative and two positive samples presented inconclusive results when assayed with TpN17. Concerning the *T. pallidum*-positive samples, TmpA exhibited the highest RI value (2.1 ± 0.6), followed by TpN17 (3.2 ± 0.55). For *T. pallidum*-negative samples, the RI value was lower for TpN17 (0.07 ± 0.02), followed by TmpA (0.1 ± 0.04).

In addition to the individual performance of each molecule, we analyzed serial and parallel diagnostic performance using two indirect IBMP-ELISAs (Table 2).

**Table 2:** Analysis of the diagnostic performance of TpN17 and TmpA antigens in the phase II study, considering serial, and parallel analyses.

Performance parameters	Serial		Parallel	
	Values	95% CI	Values	95% CI
Sensitivity (%)	91.1	86.0–94.5	97.2	93.7–98.8
Specificity (%)	99.5	97.1–99.9	100	98–100
Accuracy (%)	95.4	92.8–97.1	98.7	96.9–99.4

95% CI, confidence interval to 95%.

In the serial analysis, sensitivity was of 91.1%, specificity was 99.5%, and accuracy was 95.4%. In parallel tests, sensitivity was 97.2%, higher than in serial analysis, with no false-positive results when negative samples were tested in parallel, resulting in 100% specificity. In parallel scheme, regardless of the set of antigens used, accuracy values exceeded the 95.4% obtained in serial analysis, reaching approximately 99%.

When evaluating the performance of the two proteins according to the clinical stage, TpN17 exhibited elevated AUC (> 98%) values for primary, secondary, and early latent syphilis. TmpA achieved an outstanding AUC of 100% for primary/secondary syphilis and elevated values (99.8%) for the early latent syphilis (Figure 3).

Despite variations in sensitivity, TmpA ranged from 83.6 to 97.4%, with the highest value observed for the secondary stage. TpN17 demonstrated 100% sensitivity for primary and secondary stages and 93.2% for early latent syphilis. All clinical stages achieved a specificity of 100%. Accuracy values revealed that TmpA was highly accurate in diagnosing all clinical stages of syphilis, ranging from 95.5 to 98.6%, while TpN17 exhibited elevated accuracy in diagnostics (>98.1%) for all clinical stages. Cohen’s Kappa (*κ*) analysis showed almost perfect agreement for both TmpA and TpN17. In *T. pallidum*-positive samples, TmpA exhibited the highest RI values: 2.86 ± 1.85 (primary syphilis), 3.18 ± 0.87 (secondary syphilis), and 2.01 ± 0.73 (early latent syphilis), followed by TpN17: 3.04 ± 1.26 (primary syphilis), 3.66 ± 0.68 (secondary syphilis), and 2.84 ± 0.77 (early latent). Both proteins demonstrated a high diagnostic capacity across all clinical stages.

### 3.3 Assessment of cross-reactivity

**Figure 4:**
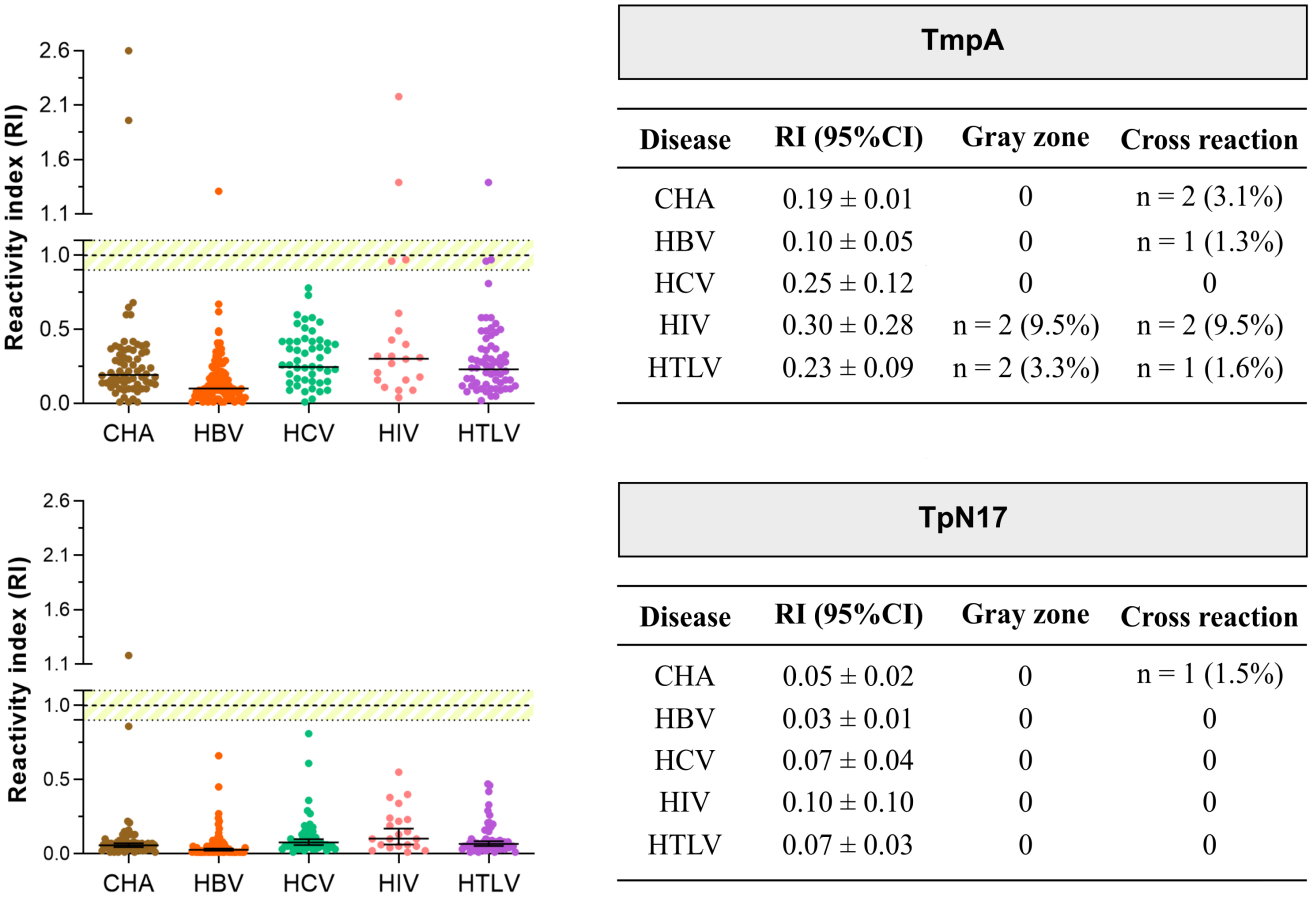
Analysis of the cross-reactivity of the *Treponema pallidum*-recombinant proteins with sera from various infectious parasitic and viral diseases. The cut-off value is reactivity index = 1.0 and the shaded area represents the grey zone (RI = 1.0 ± 0.10). CHA, chronic Chagas disease; HBV, hepatitis B; HCV, hepatitis C; HIV, human immunodeficiency virus; HTLV, human T cell lymphotropic virus; RI, reactivity index.

We employed Panel 2, consisting of 314 serum positive samples for unrelated diseases (Figure 1), to assess the cross-reactivity of antibodies against various infectious parasitic and viral diseases. As shown in Figure 4 (Supplementary Table S2), only one cross-reaction was observed (Chagas disease positive sample—1.54%) when serum samples were tested with TpN17. The incidence of cross-reactivity with TmpA antigen was also negligible, with two cases for Chagas disease (3.07%), one for HBV (1.25%), two for HIV (9.52%), and one for HTLV (1.64%) positive samples resulting in false-positive outcomes.

Considering the gray zone (RI values of 1.0 ± 0.10), no sample presented inconclusive results for TpN17, while relatively few samples were considered inconclusive for TmpA: two positive samples for HIV (9.52%), and two for the HTLV (3.3%). Regarding *T. pallidum*-positive samples with other infectious parasitic and viral diseases, TmpA exhibited the highest RI value for HIV (0.30 ± 0.28) and the lowest RI value for HBV (0.10 ± 0.05), followed by TpN17 with the highest and lowest RI values for HIV (0.10 ± 0.102) and HBV (0.03 ± 0.01), respectively.

## 4 Discussion

In this study, we evaluated the diagnostic performance of two recombinant proteins, TpN17 and TmpA, for the detection of specific anti-*T. pallidum* antibodies in the serum of syphilis-positive individuals using the ELISA diagnostic platform. Previous proteomic studies have identified several *T. pallidum* proteins with immunoreactivity and diagnostic biomarker potential (Brinkman et al., 2006; McGill et al., 2010; Kubanov et al., 2017). McGill et al. (2010) investigated the immunological response of treponemal proteins in both rabbit sera and sera from *T. pallidum*-positive individuals, identifying 29 reactive proteins, including 42-kDa (TmpA), 47-kDa (TpN47), 17-kDa (TpN17), and 15-kDa (TpN15). These proteins are encoded by genes tp0171 (TpN15), tp0435 (TpN17), tp0574 (TpN47), and tp0768 (TmpA), and they are known for their immunogenic and diagnostic relevance (Kubanov et al., 2017). Previous studies, conducted by our group, have also assessed the diagnostic performance of TpN17 and TmpA in phase I study (Silva et al., 2020).

Our results demonstrated that both TpN17 and TmpA achieved high diagnostic accuracy, with area under the curve (AUC) values exceeding 99% and accuracy values exceeding 95%. Additionally, the positive and negative likelihood ratios (LR+ and LR−) indicated significant results, further confirming the diagnostic potential of these proteins. These findings align with those from the phase I study, which reported AUC, specificity, and accuracy values above 91, 100, and 90.3%, respectively, for both proteins. However, there was a notable difference in sensitivity between phase I and II studies. In the pilot study, the sensitivity for both proteins was 69.9% (Silva et al., 2020), whereas in the present study, the sensitivity was 90.6% for TpN17 and 97.2% for TmpA. It is important to note that the phase I study had some limitations, including the use of non-treponemal tests as reference tests for sample recharacterization, which may not correlate well with treponemal tests, and the inability to use immunofluorescence across the entire serological panel. Thus, the diagnostic performance in the phase I study may have been compromised by the limitation of the reference tests. In contrast, this study applied one non-treponemal (VDRL) and two treponemal (ELISA and FTA-ABS) reference tests to multiple samples, which improved the accuracy of the results. Furthermore, the inclusion of clinically well-defined serological samples obtained from a specialized center in ISTs (CEDAP) helped address this limitation.

The antigen’s performance was evaluated in multiple studies for syphilis diagnosis, corroborating our findings. In a study by Rodríguez et al. (2002), they assessed ELISA anti-TmpA and obtained high sensitivity (93.3%) and specificity (95.3%) values. Similarly, IJsselmuiden et al. (1989) found a high specificity of 99.6% in TmpA ELISA. Martin et al. (2008) evaluated an ELISA in-house (TpN47, TpN17, TpN15, and TmpA) and demonstrated 100% of sensitivity, specificity, and accuracy for all proteins. In contrast, an rTpN17-ELISA had lower sensitivity (84.4%) but excellent specificity (100%) (Sun et al., 2009).

According to clinical stages of the infection, TmpA exhibited higher sensitivity for secondary syphilis (97.4%), following by primary syphilis (98.7%), and early latent syphilis (83.6%), while TpN17 displayed 100% sensitivity for primary and secondary syphilis and 93.2% for early latent syphilis. Both proteins showed 100% specificity across all stages. Rostopira et al. (2003) demonstrated a sensitivity of 100% for both TmpA and TpN17 among primary seropositive individuals and secondary active syphilis. For latent syphilis, TmpA obtained 87%, and TpN17 95.7% sensitivity (Rostopira et al., 2003). Furthermore, TmpA ELISA exhibited diagnostic performance of 76, 100, and 98% sensitivity for primary, secondary, and early latent syphilis, respectively (Ijsselmuiden et al., 1989).

Brinkman et al. (2006) identified variations in the humoral immune response to *T. pallidum* proteins across different clinical stages. Typically, TpN47 and flagellins (TpN37, TpN33, and TpN30) are produced in primary syphilis, while TpN47, TmpA, TpN37, TpN34.5, TpN33, TpN30, TpN17, and TpN15 are more immunogenic in secondary and early latent syphilis (Norris et al., 2001). However, ELISA’s diagnostic capacity was consistently higher in all stages, possibly due to bacterial spread (including the central nervous system) along with local replication and a robust inflammatory response (Norris et al., 2001; LaFond and Lukehart, 2006; Sandes et al., 2017). In individuals with late latent and tertiary syphilis, the humoral response is weaker, but some immunoglobulins (IgG) remain reactive to TTs compared to the NTTs (Norris et al., 2001).

Various recombinant proteins, including TpN17 and TmpA, have shown promise for syphilis serodiagnosis and are used in combination with commercial ELISA tests. Several tests have already been evaluated for their diagnostic performance: Trep-Sure (Sensitivity: 99.3–100%; Specificity: 82.6–100%) (Binnicker et al., 2011; Malm et al., 2015; Buono et al., 2017; Fakile et al., 2018; Park et al., 2019), ICE Syphilis Detection Pack (Sensibility: 100%; Specificity: 92–100%) (Aktas et al., 2007), Trep-ID (Sensitivity: 100%; Specificity: 99%) (Binnicker et al., 2011), TP-ELISA (Sensitivity: 100%; Specificity: 91.9%; Accuracy: 95.4%; AUC: 99.9%) (Liu et al., 2014), Enzywell (Sensitivity: 96.3–100%; Specificity: 92–99.7%) (Young et al., 2000; Aktas et al., 2007; Malm et al., 2015), DIALAB Syphilis (Sensitivity: 98.4%; Specificity: 94.9%) (Negash et al., 2018), RecomWell (Sensitivity: 98.9%; Specificity: 98.9%) (Sambri et al., 2001), Architect Syphilis Tp ELISA (Sensitivity: 100%; Specificity: 97.8%) (Sönmez et al., 2018), 3nd ELISA kit Dia-Syph and 2nd EIA (Sensitivity: 96.4%; Specificity: 99.7–98%) (Rostopira et al., 2003), Syphilis Screening ELISA (Sensitivity: 98.3%; Specificity: 98.7%) (Sambri et al., 2001), Eti-syphilis-G (Sensitivity: 100% for all clinical stages; Specificity: 93%) (Castro et al., 2003), Captia Select Syph-G (Sensitivity: 99%; Specificity: 98%) (Woznicová and Vališová, 2007), and others with elevated sensitivity and specificity values higher than 90% (Cole et al., 2007).

Considering our results, these commercial ELISA tests provided concordant results. Other studies have evaluated the diagnostic performance of recombinant proteins for syphilis diagnosis with values below or equivalent to our findings: such as TS-EIA (Senitivity: 52%) (Gratzer et al., 2014), Trep Check (Sensitivity: 63.4–98.9%; Specificity: 95.6–98.6%) (Tsang et al., 2007; Binnicker et al., 2011), ICE Syphilis Murex (Sensitivity: 75–98.2%; Specificity: 99.2–99.5%) (Schmidt et al., 2000; Manavi et al., 2006; Cole et al., 2007), Vircell Syphilis ELISA (Sensitivity: 73.2%; Specificity: 62.6%) (Sonmez et al., 2019), Enzygnost Syphilis (Sensitivity: 69.2–100%; Specificity: 98.5–99.8%) (Gutiérrez et al., 2000; Schmidt et al., 2000; Marangoni et al., 2009), Chorus Syphilis Screen Recombinant (Sensitivity: 87.9%; Specificity: 91.2%), Euroimmun *Treponema pallidum* screen ELISA (Sensitivity: 87.5%; Specificity: 85.7%) (Sonmez et al., 2019). Differences in performance between ELISA tests may have been influenced by various factors, including standardization, antigenic matrix, the number of samples evaluated, the analytical sensitivity, and the staging of infection.

In contrast to our findings, commercial ELISA tests using Murex ICE EIA demonstrated varying positivity rates: 84% for primary syphilis, 100% for secondary syphilis, 75% for early latent syphilis, and 100% for cases with an unknown duration (Manavi et al., 2006). Meanwhile, Owusu-Edusei et al. (2011) reported comparable results with 80% sensitivity for primary syphilis and 100% sensitivity for secondary and early latent syphilis, indicating lower sensitivity for early-stage infections. In the case of Trep-Sure, recomWell, Syphilis Screening ELISA, and Enzywell, their values consistently exceed 94% across all clinical stages, as previously documented (Young et al., 2000; Park et al., 2011, 2019, 2020).

Cross-reaction was evaluated against serological samples from individuals with unrelated diseases, including chronic Chagas disease, hepatitis B, hepatitis C, HIV-1/2, and HTLV-1/2. All samples were assessed by VDRL, ELISA, and FTA-ABS to rule out false positives due to IgG anti-*T. pallidum*. In the phase I study, cross-reactivity using TmpA was negligible, with only three positive samples (dengue, filariasis, and schistosomiasis) producing a false-positive result. No cross-reaction was observed for TpN17 in serum samples from non-bacterial infections. However, higher numbers of leptospirosis-positive samples presented positive results using TmpA (32.1%) and TpN17 (8.6%) (Silva et al., 2020). TpN17 showed less cross-reaction compared to TmpA, as observed in the phase II study. Only one cross-reaction was observed (1.49%) for the chronic Chagas disease with TpN17, while TmpA showed cross-reactions for chronic Chagas disease (2.98%), HBV (1.0%), HCV (1.4%), HIV-1/2 (1.8%), and HTLV-1/2 (1.0%) positive samples. However, non-specific reactions were observed in these and other studies but were considered diagnostically irrelevant due to the low number of false-positive samples.

Rodríguez et al. (2002) observed cross-reaction for ELISA anti-TmpA when serum samples were positive for mononucleosis, hepatitis, diabetes mellitus, HIV/AIDS, and old age. Antibodies anti-TpN17 (IgG) were recorded in 3.7% of cases in patients with gonorrhea, and IgM antibodies were detected in 2.1% of cases in the group with hepatitis C. For the TmpA protein, one false-positive result (2.5%) was found in a hepatitis B virus-infected patient (Rostopira et al., 2003). Sambri et al. (2001) evaluated 60 samples from the cross-reacting panel by recomWell Treponema IgG, and two samples were reactive: one from a Lyme disease patient and one from a patient with infectious mononucleosis. Other studies also observed cross-reaction for mononucleosis (10%) and Lyme borreliosis (13.3%) using Enzygnost Syphilis (combined proteins) (Gutiérrez et al., 2000; Marangoni et al., 2009). rTpN17-ELISA and rTpN15-17-47-ELISA returned positive results for systemic lupus erythematosus and rheumatic arthritis samples (Sun et al., 2009). Furthermore, Woznicová and Vališová (2007) used Captia Select Syph-G and obtained cross-reaction for Lyme borreliosis, chlamydial urethritis, psychosis, and allergic dermatitis.

## 5 Conclusion

In summary, both TmpA and TpN17 proteins demonstrated excellent diagnostic performance in distinguishing between positive and negative samples. Notably, the performance parameters in this assessment surpassed those observed in the phase I study. The utilization of both treponemal (ELISA and FTA-ABS) and non-treponemal (VDRL) reference tests for sample recharacterization, along with well-defined serology and clinical data, significantly contributed to these improved results. Additionally, the new standardization of experimental conditions enhanced the tests and led to advancements in the phase II study. In conclusion, our findings indicate that these proteins exhibit high diagnostic capacity, as evidenced by their specificity, sensitivity, accuracy, LR (+/−), and DOR values in ELISA. Nonetheless, we recognize the potential for further enhancement in sensitivity through the utilization of antigenic mixtures, which will be the primary focus of our group’s upcoming research endeavors.

### 5.1 Limitations

The primary limitations of this study included the absence of syphilis samples from pregnant women and cases of congenital syphilis, as well as the lack of samples from individuals with tertiary syphilis, which limited the evaluation of the diagnostic performance of both recombinant proteins. Additionally, the stratification of samples by clinical stage influenced the analysis of our results. Due to this stratification, each clinical stage had fewer samples compared to the sample size predicted by OpenEpi. Therefore, a concentration of diagnosed patients predominantly in secondary and recent latent syphilis stages limited the diversity of our sample collection. Another constraint was the inability to secure well-defined serologic samples for assessing cross-reactivity, resulting in a small number of samples being analyzed in this study, with the exclusion of *Leptospira*-positive samples.

## Data availability statement

The original contributions presented in the study are included in the article/Supplementary material, further inquiries can be directed to the corresponding author.

## Ethics statement

The studies involving humans were approved by Institutional Review Board (IRB) for Human Research at the Gonçalo Moniz Institute of the Oswaldo Cruz Foundation (IGM-FIOCRUZ), Salvador, Bahia-Brazil, under protocol number 4.581.337 (CAAE 42341121.0.0000.0040). The studies were conducted in accordance with the local legislation and institutional requirements. The participants provided their written informed consent to participate in this study.

## Author contributions

ÂS: Conceptualization, Formal analysis, Investigation, Methodology, Writing – original draft, Writing – review & editing. AL: Investigation, Methodology, Writing – original draft. LV: Investigation, Methodology, Writing – original draft. RA: Methodology, Writing – original draft. NF: Formal analysis, Investigation, Methodology, Writing – original draft. TO: Investigation, Methodology, Writing – original draft. MC: Investigation, Methodology, Writing – original draft. FM: Writing – original draft, Resources. NZ: Conceptualization, Formal analysis, Funding acquisition, Resources, Validation, Writing – review & editing. IS: Funding acquisition, Resources, Supervision, Writing – review & editing. FS: Conceptualization, Data curation, Formal analysis, Funding acquisition, Methodology, Project administration, Resources, Software, Supervision, Validation, Visualization, Writing – review & editing.

## Footnotes

^1^ https://www.openepi.com
